# Nanomedicines for Delivery of Cytarabine: Effect of Carrier Structure and Spacer on the Anti-Lymphoma Efficacy

**DOI:** 10.3390/polym17212837

**Published:** 2025-10-24

**Authors:** Robert Pola, Eliška Grosmanová, Michal Pechar, Libor Kostka, Eva Pokorná, Liliana Tušková, Pavel Klener, Tomáš Etrych

**Affiliations:** 1 Institute of Macromolecular Chemistry, Czech Academy of Sciences, Heyrovského nám. 2, 162 00 Prague, Czech Republic; 2 Institute of Pathological Physiology, First Faculty of Medicine, Charles University, Prague, U Nemocnice 5, 128 53 Prague, Czech Republicpavel.klener@gmail.com (P.K.); 3 First Department of Internal Medicine, Charles University General Hospital in Prague, Czech Republic, U Nemocnice 2, 128 08 Prague, Czech Republic

**Keywords:** HPMA copolymer, drug delivery, cytarabine, mantle cell lymphoma, nanotherapeutics

## Abstract

High-dose therapy with cytarabine (araC) is a standard treatment for aggressive non-Hodgkin lymphomas, but its efficacy is limited by rapid enzymatic degradation. To overcome this, araC was conjugated to *N*-(2-hydroxypropyl)methacrylamide (HPMA) copolymers to form linear and star-like nanomedicines using six different spacers: 3-aminopropanoyl, 5-pentanoyl, 6-aminohexanoyl, 4-aminobenzoyl, glycyl, and diglycyl. The conjugates contained 12.5–14.7 wt% araC and exhibited distinct hydrolytic release profiles at pH 7.4. **LC1** (3-aminopropanoyl) and **LC6** (diglycyl) released the drug most rapidly (~80% bound after 72 h), and **LC2**, **LC3**, and the star conjugate **SC1** showed intermediate stability (~90%), while **LC4** (4-aminobenzoyl) was most stable (~95%). In vivo, all conjugates markedly suppressed tumor growth in patient-derived xenograft models of mantle cell and Burkitt lymphoma compared with free araC. **LC1** and **LC2** provided the most durable tumor control, delaying regrowth beyond 40 days, and **SC1** achieved comparable efficacy at a reduced araC-equivalent dose (2 mg/mouse vs. 3 mg/mouse for linear conjugates). These results demonstrate that spacer structure critically influences drug release and identify **LC1** and **LC2** as promising candidates for further development in lymphoma therapy.

## 1. Introduction

The efficacy of small-molecule anticancer drugs is often limited by suboptimal pharmacokinetics, a short systemic circulation half-life, and low accumulation in target tissues. These shortcomings can be mitigated through drug delivery systems (DDSs) that address the above-mentioned limitations [1]. Various DDSs—based on organic or inorganic nanoparticles, liposomes, micelles, and water-soluble polymers—have been employed and extensively evaluated. Among these DDSs, water-soluble polymers play a significant role as highly biocompatible carriers with low accumulation in healthy tissues [2]. Consequently, macromolecular therapeutics typically reduce adverse effects and improve overall treatment efficacy. Numerous antitumor drugs (e.g., doxorubicin [3], pirarubicin [4], paclitaxel [5], cisplatin [6], 5-fluorouracil [7], and gemcitabine [8]) have been successfully conjugated to hydrophilic polymer carriers, including poly(ethylene glycol), poly(2-oxazoline)s, and copolymers based on N-(2-hydroxypropyl)methacrylamide (HPMA) [9].

Mantle cell lymphoma (MCL) is an aggressive subtype of non-Hodgkin lymphoma (NHL), accounting for approximately 6% of cases. Prognosis remains poor. Treatment paradigms have evolved over the years; current drug therapy often includes rituximab-based chemoimmunotherapy such as R-CHOP (rituximab, cyclophosphamide, doxorubicin, vincristine, and prednisone). In younger or fit patients, high-dose cytarabine-containing chemotherapy remains the standard of care for MCL [10]. A key limitation of cytarabine is its low stability in the bloodstream due to rapid enzymatic deamination by cytidine deaminase to the inactive metabolite arabinofuranosyluracil (araU) [11]. To overcome this obstacle, higher doses are administered, but systemic toxicity becomes dose-limiting.

Several drug delivery systems have been developed to improve the pharmacokinetic properties and therapeutic index of cytarabine. Liposomal formulations such as DepoCyte^®^ were designed to provide sustained drug release and prolonged cerebrospinal fluid exposure; however, their use has been limited due to severe neurotoxicity and instability during storage [12]. Nanoparticle- and micelle-based systems have also been explored to enhance araC circulation time and tumor accumulation, but they often suffer from rapid clearance by the reticuloendothelial system or premature drug release before reaching the tumor site [13,14]. Polymer–drug conjugates of cytarabine with synthetic or natural carriers, including PEG or dextran, demonstrated improved solubility and partial protection from enzymatic degradation, yet their therapeutic benefit remained modest due to insufficient stability of the linker and suboptimal control of drug release kinetics [15,16]. Therefore, more stable and biocompatible polymer–araC systems with tunable drug release remain in high demand to overcome these limitations.

Recently, we reported [17] the synthesis and preliminary biological evaluation of two HPMA copolymer-based cytarabine (araC) conjugates that differed in the spacer between the polymer chain and araC. We found that their in vivo antitumor efficacy against mantle cell lymphoma (MCL) depends on the hydrolytic stability of the amide bond between araC and the polymer carrier at physiological pH (7.4), mimicking bloodstream conditions. The drug-release rate was governed by the spacer structure. Preliminary biological data further suggested that greater conjugate stability at pH 7.4 translated into improved in vivo anti-lymphoma efficacy, most likely due to prolonged blood circulation of the polymer nanotherapeutics.

**Figure 1:**
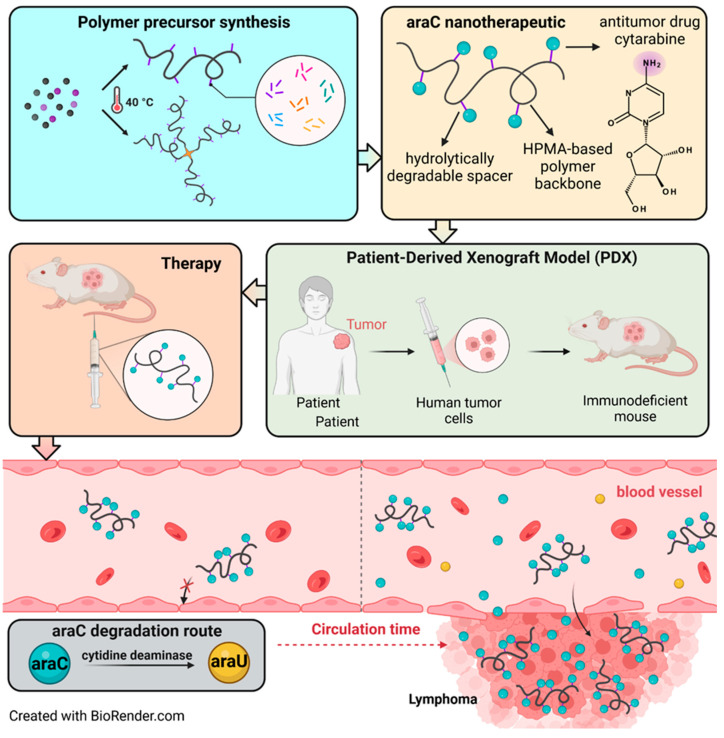
Preparation of araC-nanotherapeutic, and its fate in an in vivo PDX model.

In the present work, we extensively studied the structure-to-efficacy relationship of polymer–araC conjugates containing various spacers between the araC and the main polymer chain and employing either linear or high-molecular-weight star polymers with much prolonged blood circulation as shown in Figure 1. Indeed, we compared the properties and the biological activity of the polymer–araC conjugates in a patient-derived xenograft model of mantle cell lymphoma.

## 2. Materials and Methods

### 2.1. Materials

Methacryloyl chloride, 3-aminopropanoic acid, 5-aminopentanoic acid, 6-aminohexanoic acid, 4-aminobenzoic acid, 1,3-thiazolidine-2-thione (TT), *N*,*N*-dimethylpyridin-2 amine (DMAP), triisopropyl silane (TIPS), 1-ethyl-3-(3-dimethylaminopropyl)carbodiimide hydrochloride (EDC), *N*,*N*′-diisopropylcarbodiimide (DIC), 2-(2-cyanopropan-2-yldiazenyl)-2-methylpropanenitrile (AIBN), 2-cyanopropan-2-yl benzenecarbodithioate (DTB), 1-hydroxybenztriazol (HOBt), pentaerythritol tetrakis [2-(dodecylthiocarbonothioylthio)-2-methylpropionate], 1,3-thiazolidine-2-thione (TT), tert-butyl alcohol (t-BuOH), 1-hydroxybenzotriazole (HOBt), 1,1,1,3,3,3-hexafluoro-2-propanol (HFIP), 2-chlorotrityl chloride resin, pyridine and cytarabine were purchased from Merck, Czech Republic. 1- *N*-Ethyldiisopropylamine (DIPEA), *N*,*N*-dimethylformamide (DMF), ethyl cyanoglyoxylate-2-oxime (Oxyma), 9-fluorenylmethoxycarbonyl-glycine (Fmoc-Gly-OH), piperidine, (benzotriazol-1-yloxy)trispyrrolidinophosphonium hexafluorophosphate (PyBOP) were purchased from Iris Biotech, GmbH, Marktredwitz, Germany. Initiator 2-[(2-cyano-4-methoxy-4-methylpentan-2-yl)diazenyl]-4-methoxy-2,4-dimethylpentanenitrile (V70) was purchased from FUJIFILM Wako Pure Chemical Corporation, Neuss, Germany. Aminopropan-2-ol (AMP) was purchased from Tokyo Chemical Industry Co., Ltd., Tokyo, Japan. Methacrylic acid (MA-COOH), methanol, acetonitrile, and all other organic solvents were purchased from VWR International s.r.o., Stříbrná Skalice, Czech Republic. All chemicals and solvents were of analytical grade.

### 2.2. Methods

The synthesis and purity of monomers and release of cytarabine from the polymer conjugates were monitored by reversed-phase HPLC using Chromolith Performance RP-18e columns (100 × 4.6 mm, Merck, Darmstadt, Germany) with a linear gradient of water–acetonitrile (5–95% acetonitrile) in the presence of 0.1% TFA with a UV/VIS diode array detector (Shimadzu, Kyoto, Japan). The molecular mass of the monomers was determined using mass spectrometry performed on an LCQ Fleet mass analyzer with electrospray ionization (ESI-MS; Thermo Fisher Scientific, Inc., Waltham, MA, USA). The determination of the molecular weights and dispersity of the precursors and araC-polymer conjugates and hydrolytic degradation of star conjugate were carried out by size-exclusion chromatography (SEC) on a HPLC system (Shimadzu, Japan) equipped with UV, differential refractive index (RI), viscometric and multi-angle light scattering (LS) detectors (Wyatt Technology Corp., Santa Barbara, CA, USA) using a TSK 3000 SWXL or TSK 4000 SWXL column (Tosoh Bioscience, Tokyo, Japan) and 80% methanol, 20% 0.3 M acetate buffer pH 6.5 at a flow rate of 0.5 mL min^−1^. The refractive index increment used for the determination of molecular weight was dn/dc = 0.167 mL‧g^−1^.

The content of the thiazolidine-2-thione (TT) groups and araC was determined spectrophotometrically on a Helios Alpha UV/VIS spectrophotometer (Thermospectronic, Worcestershire, UK) using the absorption coefficients in methanol for TT (ε_305_ = 10,280 L·mol^−1^·cm^−1^) and araC (ε_301_ = 8000 L·mol^−1^·cm^−1^), respectively. Hydrodynamic radii of polymer precursors and nanotherapeutics were acquired using a Nano-ZS instrument (ZEN3600, Malvern Panalytical, Malvern, UK) in PBS buffer (concentration 2 mg‧mL^−1^) and filtered through a 0.45 μm PVDF filter. The intensity of the scattered light was detected at an angle θ = 173° with a laser wavelength of 632.8 nm. Values were determined as the mean ± SD of at least five independent measurements.

### 2.3. Synthesis of Monomers, Polymer Precursors and araC-Conjugates

The monomer *N*-(2-hydroxypropyl)methacrylamide (HPMA) was prepared as described earlier by the reaction of methacryloyl chloride with 1-aminopropan-2-ol in dichloromethane [18].

Monomers MA-prop-COOH, MA-pent-COOH, MA-hex-COOH, and MA-benz-COOH were prepared by Schotten–Baumann acylation of propionic, pentanoic, hexanoic or benzoic acid with methacryloyl chloride in aqueous alkaline medium [18]. MA-G-COOH and MA-GG-COOH were prepared by automated solid-phase peptide synthesis on 2-chlorotrityl chloride resin starting from the C-terminus using standard Fmoc procedures.

3-(*N*-Methacrylamidopropanoyl)thiazolidine-2-thione (MA-prop-TT) was synthesized by the reaction of methacrylamidopropanoic acid with TT in the presence of DMAP using EDC, allowing the removal of the water-soluble urea derivative by extracting the organic solution with water. 3-(5-Methacrylamidopentaoyl)thiazolidine-2-thione (MA-pent-TT), 3-(6 methacrylamidohexanoyl)thiazolidine-2-thione (MA-hex-TT), 3-(4-methacrylamidobenzoyl)thiazolidine-2-thione (MA-benz-TT), 3-(*N*-methacryloylglycyl)thiazolidine-2-thione (MA-G-TT) and 3-(*N*-methacryloylglycylglycyl)thiazolidine-2-thione (MA-GG-TT) were prepared analogously as described above for MA-prop-TT [19]. The monomers were characterized using HPLC (single peak) and ESI-MS (MA-prop-TT: calculated 258.05, found 281.18 [M+Na] (see Supplementary Figure S1); MA-pent-TT: calculated 286.06, found 309.21 [M+Na]; MA-hex-TT: calculated 300.10, found 323.18 [M+Na]; MA-benz-TT: calculated 306.05, found 328.99 [M+Na]; MA-G-TT: calculated 244.03, found 245.11 [M+H]; MA-GG-TT: calculated 301.08, found 302.10 [M+H]).

Polymer precursors **LP1**–**LP6** (Table 1) were prepared by a reversible addition-fragmentation chain transfer (RAFT) copolymerization of HPMA with the corresponding monomer [20] as shown in Supplementary Figure S2.

Star-shaped polymer precursor **SP1** with TT groups was prepared by RAFT copolymerization of HPMA and MA-hex-TT comonomer using V-70 as an initiator and pentaerythritol tetrakis [2-(dodecylthiocarbonothioylthio)-2-methylpropionate] as a chain-transfer agent (CTA). Example of polymerization of star-shaped precursor poly(HPMA-*co*-MA-hex-TT): HPMA and MA-hex-TT with V-70 and pentaerythritol CTA were used in molar ratios of monomer: CTA: initiator 800:1:1. The molar ratio of HPMA to MA-hex-TT in the reaction mixture was 88:12. An example of the synthesis is as follows: HPMA (1.2 g, 8.3 mmol) was dissolved in 10.1 mL of tert-butyl alcohol and mixed in a polymerization ampule with MA-hex-TT (0.343 g, 1.14 mmol), CTA (18.1 mg, 11.9 µmol) and initiator V-70 (7.4 mg, 23.8 µmol) dissolved in 3.5 mL of anhydrous DMA. The polymerization mixture was bubbled with argon for 10 min and sealed. Copolymerization was carried out at 30 °C for 72 h. The copolymer was isolated by precipitation in a mixture of acetone and diethyl ether (2:1), filtered off, and dried under a vacuum. Ending DTB groups from the polymer precursor were removed by the reaction with an excess of AIBN in DMA (80 °C, 3 h) [21]. The copolymer was isolated by precipitation in a mixture of acetone and diethyl ether (1:1), filtered off, and dried under a vacuum to yield 1.2 g (78%).

**Figure 2:**
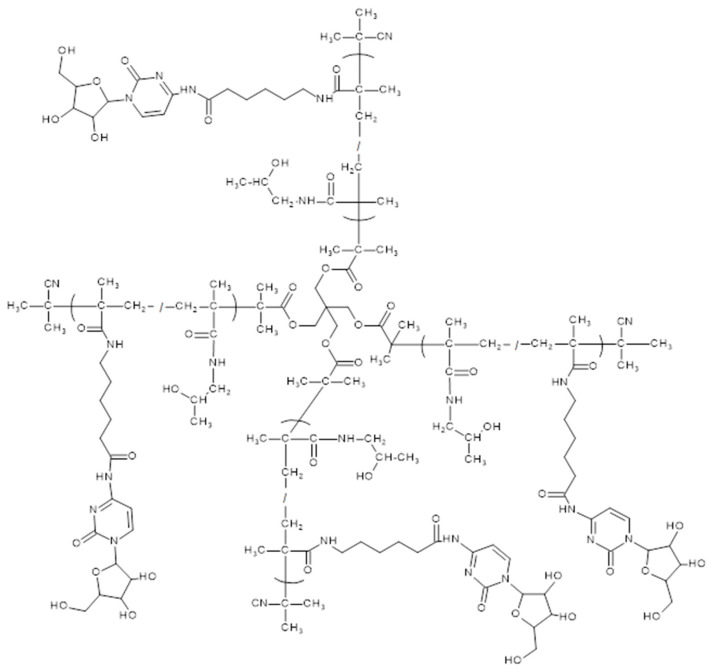
Chemical structure of star polymer conjugate **SC1** with cytarabine with hydrolytically cleavable linkers between the polymer backbone and cytarabine (araC).

The conjugation of the reactive copolymer precursors with araC in pyridine, affording conjugates **LC1**–**LC6** and **SC1** (Table 2, Supplementary Figure S2, Figure 2), was carried out similarly as described earlier [20]. Final araC-polymer nanotherapeutics were isolated from the reaction mixture by precipitation in diethyl ether and purified on a Sephadex G25 in water and freeze-dried.

### 2.4. In Vitro Release of araC from the Polymer Conjugates in PBS Buffer

The hydrolytic release of cytarabine was measured in PBS buffer at pH 7.4, to mimic the bloodstream, at 37 °C. Conjugates (2 mg‧mL^−1^) were incubated in the buffer; the mixture was measured using HPLC to observe the decrease in the absorbance at 301 nm of the polymer peak corresponding to the decrease in the content of araC on the polymer.

### 2.5. In Vitro Release of araC from the Polymer Conjugates in Serum

The hydrolytic release of cytarabine from the polymer conjugates was evaluated in human serum at 37 °C. Conjugates were incubated at a concentration of 4 mg·mL^−1^, and at designated time points, 15 µL of the incubation mixture was withdrawn and mixed and shaken with 30 µL of methanol (MeOH) to precipitate serum proteins. The resulting mixture was centrifuged, and the supernatant was analyzed by HPLC to monitor the release of free araC. The detection was performed at 278 nm, and the concentration of free araC was quantified using a calibration curve constructed from known standards of free araC.

### 2.6. Mouse Model

The PDX models VFN-D1 and VFN-B2 were established by sub-renal xenotransplantation of primary lymphoma cells obtained from a patient with chemotherapy-refractory diffuse large B-cell lymphoma (DLBCL) and Burkitt lymphoma, resp., after failure of front-line immunochemotherapy as part of other research projects approved by the Ethics Committee of the General University Hospital Prague under number 48/18 Grant AZV VES 2019 VFN. The mutational profiling implemented by whole exome sequencing (WES) confirmed that VFN-D1 and VFN-B2 cells shared the majority of somatic mutations with the primary lymphoma cells from which they were derived [22,23].

NOD·C mice (further NSG mice) purchased from the Jackson Laboratory (Bar Harbor, ME, USA) were used for all in vivo experiments. Animals were housed and maintained in a pathogen-free environment in individually ventilated cages and provided with sterilized food and water. The experimental design was approved by the institutional animal care and use committee (MSMT-8820/2022-3). Mice were xenografted with 5 million PDX cells per mouse. When mice developed palpable tumors (=day 1, D1), the mice were stratified into treatment cohorts so that each cohort contained mice with tumors of comparable size. The therapy was administered intraperitoneally (IP) on D1. The mice were carefully observed and tumors measured on working days in three perpendicular directions using a digital caliper. Individual mice were euthanized when tumors reached 2 cm in the largest diameter. Growth curves were calculated using the following formula: π/6 × length × width × height.

## 3. Results and Discussion

**Figure 3:**
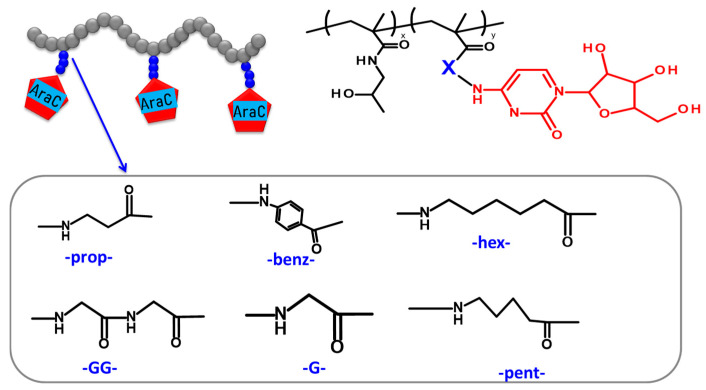
Schematic and general chemical structure of polymer conjugates with cytarabine with hydrolytically cleavable linkers between the polymer backbone and cytarabine (araC).

In the present study, HPMA copolymer-based nanotherapeutics (Table 2) containing covalently bound cytostatic drug araC were designed, synthesized, and evaluated for their in vivo biological behavior, anti-lymphoma efficacy in a murine patient-derived xenograft (PDX) model, and potential toxicity. AraC was attached to the polymer backbone via a series of spacers (Figure 3) whose structure determined the hydrolysis rate of the amide bond between the araC and polymer carrier. The nanotherapeutics are designed to circulate in a stable form in the bloodstream, to be preferentially accumulated within the tumor mass and, consequently, to release the majority of the drug in the tumor. Moreover, covalent attachment of the drug to the polymer carrier prevents undesired deamination of araC to its inactive derivative, araU, occurring during the standard therapy with the free drug.

### 3.1. Synthesis and Physico-Chemical Characterization of the Polymer Precursors and Conjugates

**Figure 4:**
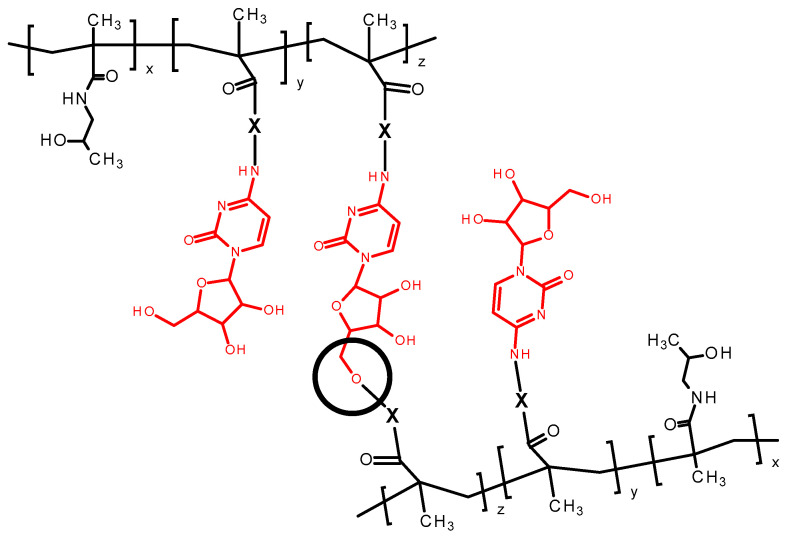
Scheme of possible crosslinking of the primary hydroxy group of araC after reaction with the multi-valent copolymer precursors.

All copolymer precursors were prepared by a controlled RAFT polymerization technique to guarantee the maximal uniformity of the synthesized polymer-drug conjugates and to ensure the batch-to-batch reproducibility. All linear copolymer precursors had weight-average molecular weight (Mw) between 35,000 and 45,000 g‧mol^−1^, dispersity below 1.2 and the polymerization degrees ranging from 219 to 277. In the case of **SP1**, which is composed of four polymer chains, the average degree of polymerization is 198 monomer units per chain. The polymer precursor displayed characteristic absorption maxima at 272 and 306 nm in the UV/VIS spectrum, whereas conjugation with AraC resulted in a spectral shift with maxima at 246 and 301 nm, thereby confirming the successful attachment of the drug as shown in Supplementary Figure S5. Conjugation of araC to the polymer precursors led to a slight molecular weight increase and a broadening of dispersity in the resulting polymer nanotherapeutics, as compared to Table 1 and Table 2. The observed increase in molecular weight and dispersity (as shown in Supplementary Figure S4) is caused by partial acylation of the primary hydroxyl group of the arabinose moiety of araC after reaction with the multivalent copolymer precursors containing reactive TT groups (see Figure 4), which resulted in partial branching of the polymer chains. As previously reported [17], upon incubation in aqueous medium at pH 7.4, the molecular weight of polymer–araC conjugates gradually decreased to the value of the polymer precursor, indicating hydrolysis of the ester bonds of the acylated arabinose moieties and release of araC. An increase in molecular weight during araC attachment to **SP1** was also observed. The final **SC1** conjugate, despite an approximately fourfold increase in Mw during araC attachment, was fully soluble in buffer and was used in the subsequent biological tests.

Importantly, the polymer precursors **LP1**–**LP6** were designed to have molecular weights and hydrodynamic radii below the renal threshold, making them readily excretable from the organism via renal filtration. In contrast, the corresponding polymer–drug conjugates **LC1**–**LC6** exhibited molecular parameters that led to prolonged blood circulation and, consequently, to improved therapeutic activity compared with free araC. Nevertheless, after araC release, the polymer carrier, having a molecular weight below the renal threshold limit, will be rapidly eliminated. We are convinced that the improved pharmacokinetics of the polymer–araC conjugates, determined by their hydrodynamic size and excellent biocompatibility, is the key factor responsible for their enhanced antitumor activity compared with free araC. To verify whether a further increase in size would lead to improved anti-lymphoma efficacy, we designed and synthesized the star polymer precursor **SP1** and the corresponding polymer–drug conjugate **SC1**, with molecular parameters (Mw = 470,000 g·mol^−1^; D_h_ = 27.8 nm) significantly exceeding the renal threshold. The star precursor structure was designed to be biodegradable, as the ester bonds incorporated into the core of the star polymer are expected to undergo hydrolytic degradation under neutral aqueous conditions. After hydrolytic cleavage of the star polymer, the resulting degradation products are anticipated to be ultimately eliminated from the organism via glomerular filtration.

### 3.2. Hydrolytic Release of araC from the Polymer Conjugates in PBS Buffer

**Figure 5:**
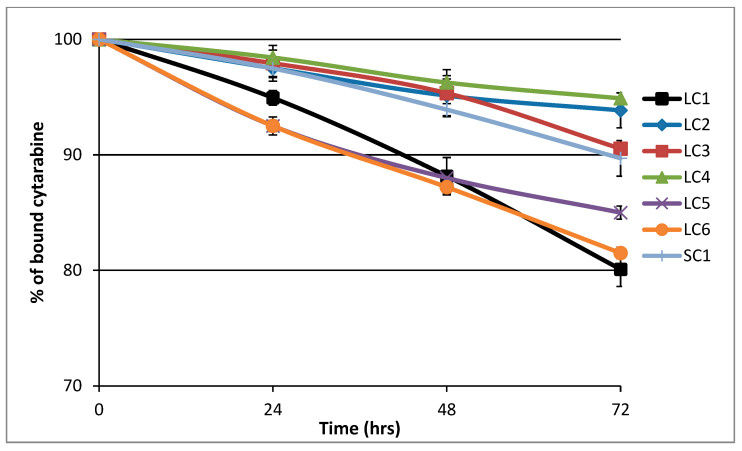
Hydrolytic release of araC from conjugates at pH 7.4. The release of free araC was monitored at 301 nm from the decreasing peak of the polymer-bound araC using HPLC.

All polymer–araC conjugates were incubated in PBS buffer at pH 7.4 in order to determine the rate of hydrolytic drug release under conditions mimicking the situation after polymer nanotherapeutic administration (see Figure 5). The highest rate of araC release was observed for conjugates **LC1**, **LC5**, and **LC6**, which contained 3-aminopropanoyl, glycyl, and diglycyl spacers, respectively. The release of the drug was slower when 6-aminohexanoyl or 5-pentanoyl spacers were employed in **LC3** and **LC2**, respectively. Interestingly, the slowest drug release was found for conjugate **LC4** with a 4-aminobenzoyl spacer, which exhibited release kinetics comparable to those of **LC2** containing the 5-pentanoyl spacer. No significant difference in araC release was observed between the conjugates containing the 6-aminohexanoyl spacer—namely, the linear polymer **LC3** and the star polymer **SC1**—indicating that the spacer structure primarily governs the rate of hydrolytic araC release.

These results indicate that the major factor influencing the rate of drug release is the hydrophobic nature of the spacer between the polymer chain and araC. We hypothesize that a more hydrophobic spacer adjacent to the amide bond of araC may reduce water accessibility to the bond and thereby slow its hydrolysis. Importantly, the spacer structure influences the drug release rate much more significantly than the size of the polymer conjugate.

### 3.3. Hydrolytic Release of araC from the Polymer Conjugates in Human Serum

**Figure 6:**
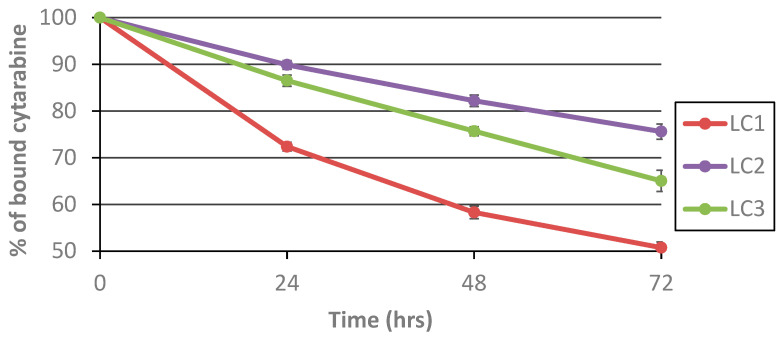
Hydrolytic release of araC from three conjugates in human serum. The release of free araC was monitored at 278 nm from the increasing peak of the free araC using HPLC.

In order to evaluate the stability of the polymer conjugates in a more physiologically relevant environment, araC release experiments were performed in human serum. Three polymer–araC conjugates were selected based on their hydrolytic release profiles: one from the slowest-releasing group (**LC2**), one from the fastest-releasing group (**LC1**), and one exhibiting intermediate release kinetics (**LC3**). All three conjugates were incubated in human serum at 37 °C to assess and compare their drug release rates under biologically relevant conditions (see Figure 6).

Incubation in human serum accelerated the release rate of araC for all polymer conjugates. We assume that this acceleration may be caused by the presence of serum enzymes capable of catalyzing slow amide bond cleavage, such as arylamidases, carboxylesterases, and butyrylcholinesterase, which are known to exhibit weak amidase or peptidase activity in plasma [24,25,26]. Even in human serum, however, the slowest araC release was observed for **LC2**, with approximately 25% of the drug released after 3 days of incubation, demonstrating that this polymer system is the most stable conjugate under blood-mimicking conditions. **LC3** showed a slightly higher release rate (35% after 3 days), while **LC1** exhibited the fastest hydrolysis, with about 50% of araC released after 3 days. The calculated half-lives of araC release in human serum are summarized in Table 3.

In both environments, **LC1** exhibited the fastest release, followed by **LC3**, whereas **LC2** displayed the slowest release profile. This result confirms the chemical stability of the linkers under physiological pH in a non-enzymatic setting. In contrast, incubation in human serum resulted in markedly accelerated drug release, highlighting the influence of enzymatic and protein-mediated cleavage mechanisms. These findings demonstrate that interactions with enzymes and serum proteins significantly enhance linker cleavage. The comparison clearly indicates that the release behavior observed in PBS may underestimate the actual cleavage occurring in biological systems. Therefore, evaluation in serum or plasma is essential for a more accurate prediction of in vivo performance.

### 3.4. Hydrolytic Degradation of the Star Polymer Conjugate

To verify the degradation of the star polymer conjugate **SC1**, the conjugate was incubated in PBS buffer (pH 7.4) mimicking bloodstream conditions, and its molecular weight was monitored by SEC over a 28-day period.

**Figure 7:**
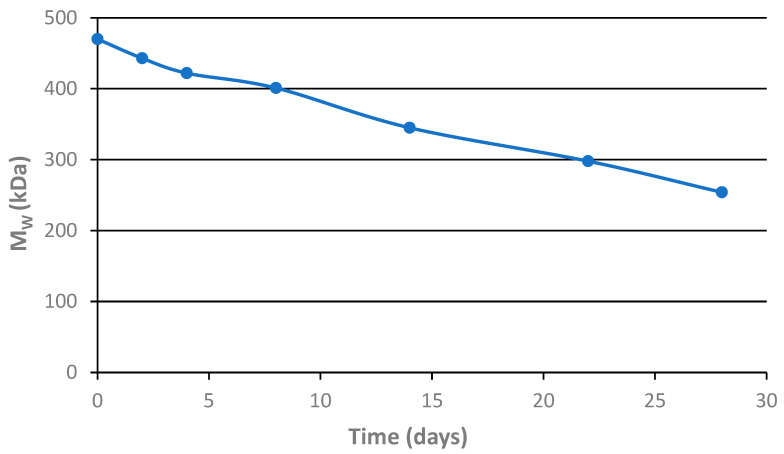
Hydrolytic degradation of the star conjugate **SC1** in PBS buffer at pH 7.4. after 2, 4, 8, 14, 22, 28 days. The Mw of the main polymer fraction is depicted.

The molecular weight of the main polymer fraction, as shown in Figure 7, clearly demonstrates that the molecular weight of the star polymer conjugate gradually decreases during incubation. Moreover, the SEC chromatograms reveal the formation of an additional peak corresponding to the size of the polymer arms bound to the star polymer core. Although the observed degradation rate under the experimental conditions was relatively low, we are convinced that hydrolysis under actual in vivo conditions will proceed more rapidly and will ultimately yield degradation products that can be excreted from the organism via glomerular filtration.

### 3.5. In Vivo Efficacy and Toxicity in VFN-D1 DLBCL Model

Monitoring of body weight in VFN-D-bearing mice during treatment with araC-based linear conjugates **LC1**, **LC2**, and **LC3** provided important insights into the tolerability and systemic toxicity of these experimental therapies. All treatment groups received the same dose (3 mg araC equivalent per mouse), and body weights were recorded throughout the 67-day study (see Supplementary Figure S6). Importantly, none of the treatment groups exhibited rapid or sustained weight loss exceeding 15–20%, which would suggest severe toxicity. Therefore, all three formulations appeared to be generally well tolerated, although **LC2** may require further investigation for potential formulation-related adverse effects. Overall, the body weight monitoring data support the continued development of **LC1** and **LC3** as efficacious and well-tolerated nanotherapeutics and highlight the importance of comprehensive in vivo safety assessment alongside therapeutic efficacy.

**Figure 8:**
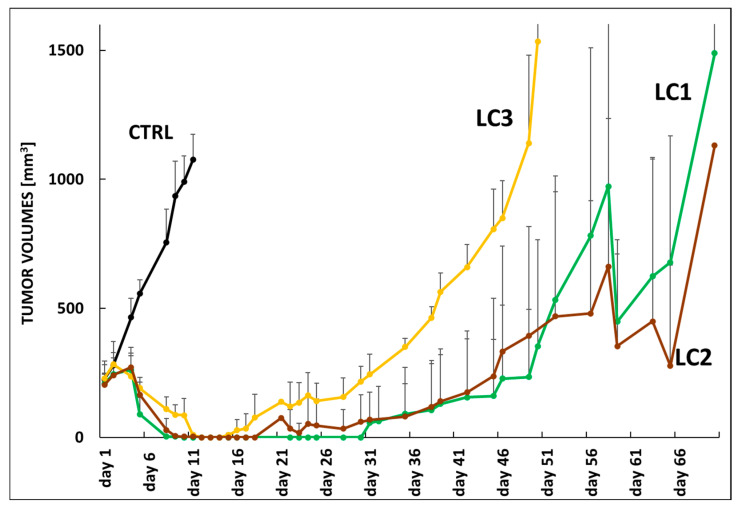
Experimental therapy of KTC-bearing mice with linear conjugates **LC1**, **LC2** and **LC3**. Mice were xenografted with the PDX cells derived from a patient with relapsed mantle cell lymphoma (KTC). Therapy was administered IP on day 1, when all mice developed tumors with a calculated volume of approx. 250 mm^3^. Each cohort contained 5 animals. The following dosing was used: **LC1** (green line) 3 mg araC eq./mouse, **LC2** (brown line) 3 mg araC eq./mouse, **LC3** (yellow line) 3 mg araC eq./mouse. The results were compared to untreated animals (CTRL, black line).

Based on the tumor growth curves shown in Figure 8, the antitumor effects of the three linear conjugates (**LC1**, **LC2**, and **LC3**) were compared in a relapsed mantle cell lymphoma PDX model (KTC) in immunodeficient mice. The aim was to investigate how the spacer structure and the corresponding araC release rate influence the overall therapeutic efficacy of the conjugates. The conjugates were administered intraperitoneally on day 1 (3 mg araC equivalent per mouse), when all mice exhibited tumors of approximately 250 mm^3^ in volume. The untreated control group (CTRL, black line) showed rapid and continuous tumor progression, reaching volumes above 1000 mm^3^ by day 11, clearly indicating the aggressive nature of the model and the necessity for intervention. In contrast, all treated groups demonstrated an initial tumor regression phase (days 1–11), indicating strong early therapeutic activity. Among the tested groups, **LC1** (green) and **LC2** (brown) exhibited the strongest anti-lymphoma efficacy, with tumor volumes remaining significantly lower than those of the CTRL and **LC3** groups throughout the observation period. **LC1** displayed slightly higher variability, but both **LC1** and **LC2** effectively delayed tumor regrowth until approximately day 46, after which tumors began to increase gradually. **LC3** (yellow) demonstrated an intermediate effect, with partial tumor control followed by steady regrowth starting around day 21. By day 51, **LC3** reached tumor volumes comparable to those observed for **LC1** and **LC2** on day 71. Considering the half-lives of araC release in serum (ranging from 3 to 6 days depending on the conjugate), it appears that the initial therapeutic activity of the conjugates remained relatively similar up to day 16, when the first signs of divergence in treatment efficacy became apparent. It can be hypothesized that **LC1**, which exhibited a somewhat higher araC release rate, produced the most effective suppression of tumor growth. Therefore, the optimal therapeutic effect likely depends not on the slowest possible release but on an appropriate, balanced release rate similar to that of **LC1**. To achieve complete tumor eradication, an additional dose of the polymeric conjugate around day 16 could be advantageous.

Overall, these data suggest that **LC1** and **LC2** exhibit superior long-term tumor control in this model compared with **LC3** and untreated animals, despite all groups receiving the same araC-equivalent dose.

### 3.6. In Vivo Efficacy in VFN-B2 Lymphoma Model

Similarly, the efficacy of the same linear conjugates (**LC1**, **LC2**, and **LC3**), differing in the spacer structure between the polymer backbone and araC, was evaluated in another PDX model of relapsed Burkitt lymphoma (VFN-B2). Treatment was initiated on day 1, when all mice exhibited established tumors with an average volume of approximately 350–400 mm^3^.

**Figure 9:**
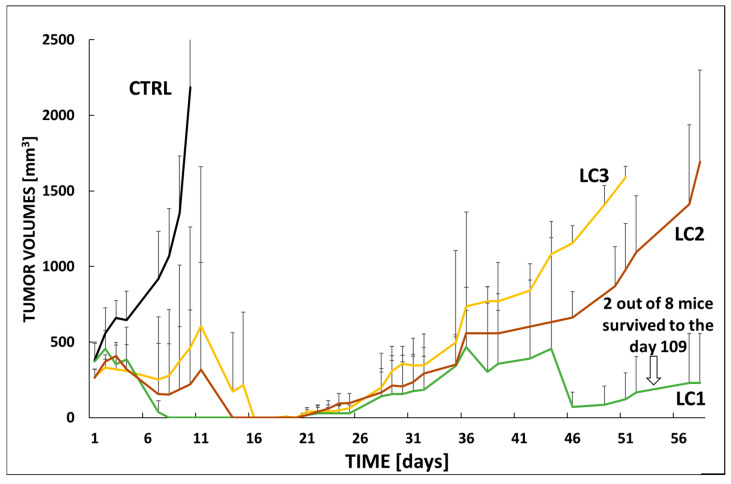
Experimental therapy of VFN-B2-bearing mice with linear conjugates **LC1**, **LC2** and **LC3**. Mice were xenografted with the PDX cells derived from a patient with relapsed mantle cell lymphoma (VFN-B2). Therapy was administered IP on day 1, when all mice developed tumors with a calculated volume of approx. 400 mm^3^. Each cohort contained 8 animals. The following dosing was used: **LC1** (green line) 3 mg araC eq./mouse, **LC2** (brown line) 3 mg araC eq./mouse, **LC3** (yellow line) 3 mg araC eq./mouse. The results were compared to untreated animals (CTRL, black line).

As expected, the untreated control group (CTRL, black line) exhibited rapid and aggressive tumor progression, with tumor volumes exceeding 2000 mm^3^ by day 11, underlining the high malignancy of the VFN-B2 model (see Figure 9). In contrast, all treated groups initially demonstrated tumor regression, similar to that observed in the VFN-D1 model. **LC1** treatment led to long-term tumor suppression in a subset of animals, with notably lower tumor volumes maintained throughout the 56-day observation period. Importantly, two out of eight mice in the **LC1** group survived beyond day 109, indicating a significant therapeutic benefit and the potential for long-term disease control.

**LC2** (brown line) and **LC3** (yellow line) also exhibited antitumor activity, although to a lesser extent. Both groups showed partial tumor growth inhibition, with **LC3** ultimately displaying the least durable response. After 36 days, tumors in the **LC3** group began to regrow faster than those in the **LC2** group. By day 51, tumor volumes in the **LC3** group had reached levels comparable to those in the **LC2** group on day 56. The gradual loss of efficacy observed in **LC2** and **LC3** suggests differences in pharmacokinetics, drug release kinetics, or tumor penetration compared with **LC1**. The results obtained in the VFN-B2 model further confirmed the previous hypothesis that **LC1** possesses an optimal araC release rate, resulting in excellent therapeutic efficacy and prolonged suppression of tumor regrowth. Even in the VFN-B2 model, complete remission could likely be achieved by administering an additional dose of the conjugates around day 16 of treatment.

Taken together, these results demonstrate that while all conjugates exhibited strong initial antitumor effects, **LC1** was clearly superior in terms of long-term tumor control and overall survival benefit, not only in the treatment of relapsed mantle cell lymphoma but potentially also in other aggressive B-cell malignancies.

### 3.7. In Vivo Efficacy Star Conjugate SC1 in VFN-B2 Lymphoma Model

**Figure 10:**
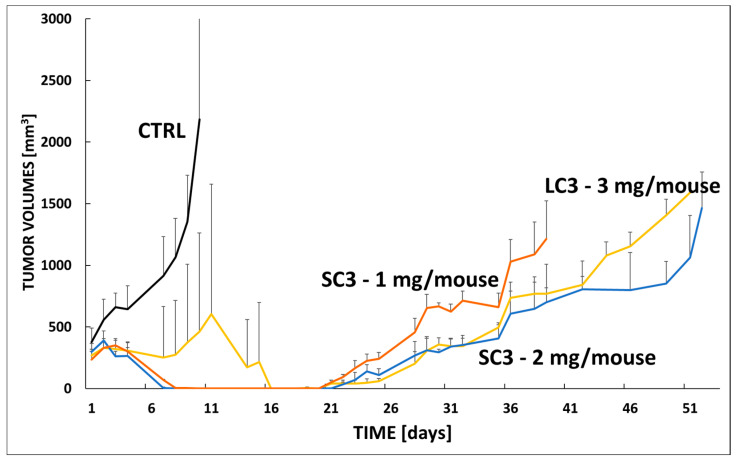
Experimental therapy of VFN-B2-bearing mice with linear conjugate **LC3** and star conjugate **SC1**. Mice were xenografted with the PDX cells derived from a patient with relapsed mantle cell lymphoma (VFN-B2). Therapy was administered IP on day 1, when all mice developed tumors with a calculated volume of approx. 400 mm^3^. Each cohort contained 8 animals. The following dosing was used: **LC3** (yellow line) 3 mg araC eq./mouse, **SC1** (blue line) 2 mg araC eq./mouse, **SC1** (orange line) 1 mg araC eq./mouse. The results were compared to untreated animals (CTRL, black line).

Finally, the antitumor efficacy of two araC-based polymer conjugates differing in carrier architecture—a linear conjugate (**LC3**) and a star-shaped conjugate (**SC1**)—was evaluated in a PDX model of relapsed mantle cell lymphoma (VFN-B2) with established tumors (300–400 mm^3^) (see Figure 10).

As expected, the untreated control group (CTRL, black line) exhibited rapid and uncontrolled tumor growth, with tumor volumes exceeding 2500 mm^3^ by day 11. Among the treated groups, **SC1** administered at 2 mg per mouse (blue line) exhibited long-term efficacy comparable to that of **LC3** administered at 3 mg per mouse (yellow line), despite the lower araC-equivalent dose. Tumor regrowth in all treated groups began after day 21 and progressed at a similar rate throughout the remainder of the observation period. This finding suggests that **SC1** may represent a more efficient drug delivery system, achieving comparable therapeutic efficacy at a lower dose. Importantly, no significant body weight loss was observed in either **SC1** treatment group.

Interestingly, the **SC1** group treated at 1 mg per mouse (orange line) initially mirrored the antitumor effect observed in the higher-dose **SC1** group but exhibited slightly accelerated tumor regrowth from day 26 onward. Nevertheless, both **SC1**-treated groups showed markedly improved tumor control compared with untreated animals, confirming the dose-dependent activity of the **SC1** system.

In conclusion, the star-shaped **SC1** conjugate demonstrated promising antitumor activity in the VFN-B2 model, particularly at the 2 mg dose, where it achieved efficacy comparable to that of the linear **LC3** conjugate administered at a higher dose. Nevertheless, considering the results obtained across both tumor models, the linear **LC1** conjugate appears to be the most effective system, exhibiting the highest antitumor activity at an equivalent dosage.

## 4. Conclusions

In summary, this study demonstrates the successful design, synthesis, and comprehensive evaluation of a series of HPMA-based polymer–AraC conjugates featuring various biodegradable linkers tailored for controlled drug release. The conjugates contained 12.5–14.7 wt% of covalently bound AraC, providing a sufficient drug payload while maintaining excellent solubility and biocompatibility. The hydrolytic release profiles in PBS and human blood serum highlighted the critical role of spacer structure in modulating drug liberation, with significantly accelerated cleavage in biologically relevant conditions. In vivo experiments in two PDX models of relapsed mantle cell lymphoma confirmed the superior antitumor efficacy and tolerability of selected conjugates, particularly **LC1**, when administered at doses equivalent to 3 mg araC/mouse. Importantly, the star-shaped conjugate **SC1** achieved comparable efficacy to the linear systems even at reduced doses of 1–2 mg araC/mouse, reflecting improved delivery efficiency. The results validate the therapeutic potential of polymer-bound araC for overcoming limitations of conventional chemotherapy, such as rapid clearance and enzymatic deactivation, and underscore the promise of polymer therapeutics for the treatment of aggressive and drug-resistant lymphomas. By adjusting the dosing regimen, this polymeric nanotherapeutic could become a very active system for the effective treatment of relapsed lymphomas.

## Figures and Tables

**Table 1:** Characterization of the polymer precursors.

Sample	Description	*M*_W_ ^a^ [g‧mol^−1^]	*Ð* ^a^ [-]	*D*_h_ ^a^ [nm]	TT Content ^b^ [mol%]	Degree of Polymerization ^c^
**LP1**	p(HPMA-*co*-MA-prop-TT)	37,100	1.07	8.8	14.0	233
**LP2**	p(HPMA-*co*-MA-pent-TT)	34,500	1.08	9.0	10.0	219
**LP3**	p(HPMA-*co*-MA-hex-TT)	44,500	1.12	8.6	11.3	277
**LP4**	p(HPMA-*co*-MA-benz-TT)	43,000	1.16	9.2	9.8	270
**LP5**	p(HPMA-*co*-MA-G-TT)	40,000	1.12	8.8	10.9	260
**LP6**	p(HPMA-*co*-MA-GG-TT)	42,000	1.15	9.0	11.8	259
**SP1**	STAR-p(HPMA-*co*-MA-hex-TT)	125,000	1.09	14.4	10.4	198/chain

^a^ Molecular weights were determined by SEC using RI and LS detection. ^b^ TT groups were determined by UV/VIS spectrophotometry in methanol (ε_305_ = 10,280 L·mol^−1^·cm^−1^). ^c^ Degree of polymerization calculated as the ratio between the molecular weight of its repeating unit with the total molecular weight of the polymer.

**Table 2:** Characterization of the polymer conjugates.

Sample	Description	*M*_W_ ^a^ [g‧mol^−1^]	*Ð* ^a^ [-]	*D*_h_ ^b^ [nm]	araC Content ^c^ [wt%]
**LC1**	p(HPMA-*co*-MA-prop-araC)	65,000	1.50	13.4	12.5
**LC2**	p(HPMA-*co*-MA-pent-araC)	53,100	1.30	15.0	13.1
**LC3**	p(HPMA-*co*-MA-hex-araC)	61,800	1.32	16.6	14.2
**LC4**	p(HPMA-*co*-MA-benz-araC)	62,800	1.35	10.4	13.2
**LC5**	p(HPMA-*co*-MA-G-araC)	62,600	1.39	10.6	13.5
**LC6**	p(HPMA-*co*-MA-GG-araC)	63,300	1.35	11.0	13.9
**SC1**	STAR-p(HPMA-*co*-MA-hex-araC)	470,000	3.9	27.8	14.7

^a^ Molecular weights were determined by SEC using RI and LS detection. ^b^ Hydrodynamic diameter was determined by a Nano-ZS instrument in PBS buffer (concentration 2 mg‧mL^−1^). ^c^ AraC was determined by UV/VIS spectrophotometry in methanol (ε_301_ = 8000 L·mol^−1^·cm^−1^).

**Table 3:** Theoretical half-time of araC—calculated time required for the concentration of araC to decrease to half of its initial amount in the polymer conjugate.

Sample	t_50_ (h)
**LC1**	72
**LC2**	146
**LC3**	100

## Data Availability

The data used to support the findings of this study are available from the corresponding author upon request due to privacy reasons.

## Supplementary Materials

The following supporting information can be downloaded at: https://www.mdpi.com/article/10.3390/polym17212837/s1, Figure S1. The spectrum of molecular mass of Ma-prop-TT [281.2 M+Na] determined by ESI mass spectrometry. Figure S2. Scheme of preparation of polymer precursor LP1 and the attachment of araC to form the conjugate LC1. Figure S3. NMR spectra of Ma-prop-OH, Ma-prop-TT, polymer precursor LP1 and the conjugate LC1. Figure S4. SEC chromatograms (differential refractive index-dRI) of LP1 (red) and LC1 (blue) measured on a TSK 3000 SWXL column (Tosoh Bioscience, Japan) in 80% methanol, 20% 0.3 M acetate buffer pH 6.5 at a flow rate of 0.5 ml min^−1^. Figure S5. UV/VIS spectra of the polymer precursor LP1 with two characteristic absorption maxima for TT at 272 and 306 nm (A), and conjugate LC1 with absorption maxima at 246 and 301 nm characteristic for attached araC (B). Figure S6. Weight of animals during the experimental therapy of KTC-bearing mice with linear conjugates LC1, LC2 and LC3.
