# Supplementary material for: Nanomedicines for Delivery of Cytarabine: Effect of Carrier Structure and Spacer on the Anti-Lymphoma Efficacy
Source: Polymers (Basel). 2025 Oct 24;17(21):2837. doi: 10.3390/polym17212837 (PMC12610423; doi:10.3390/polym17212837)
Supplement: Supplementary file 1 [file polymers-17-02837-s001.zip › polymers-3898676-supplementary.pdf]

Supporting information

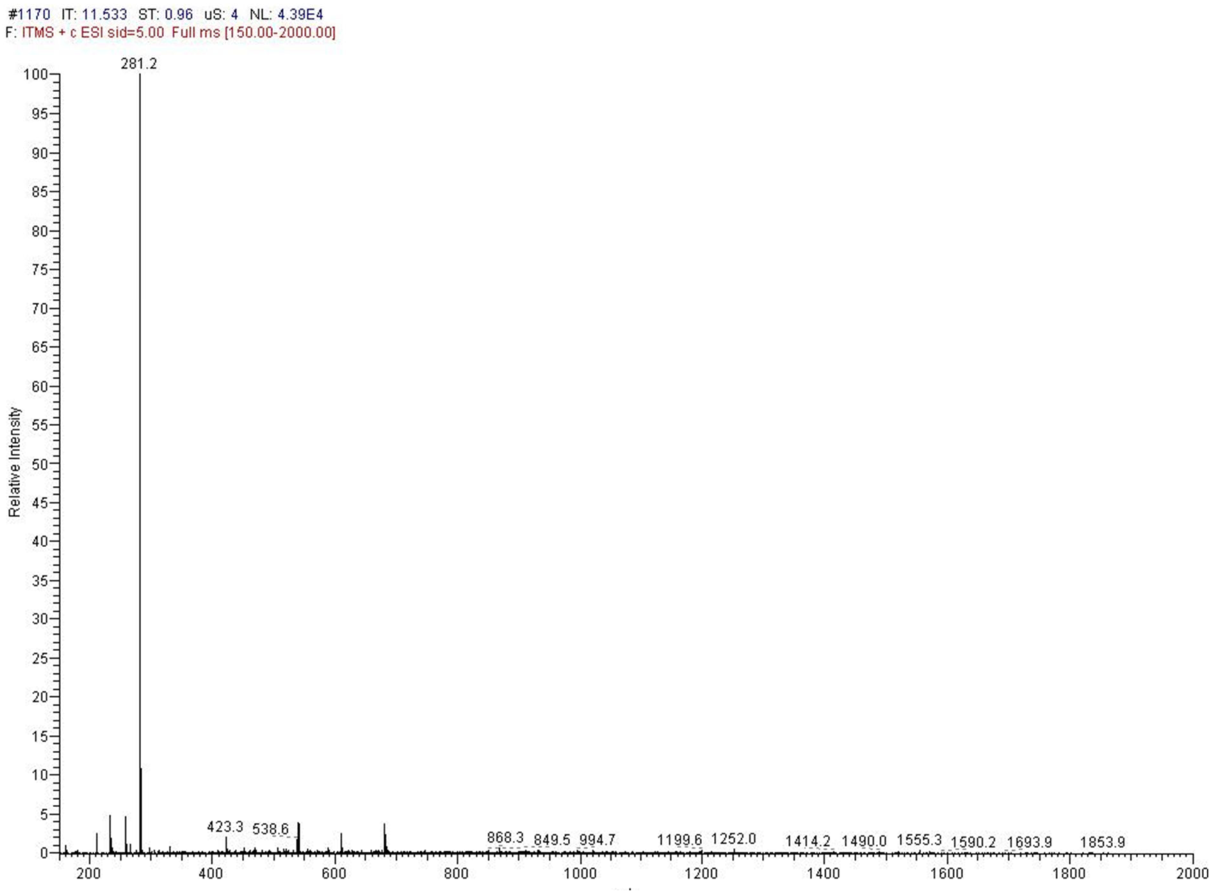

Figure S1. The spectrum of molecular mass of **Ma-prop-TT** [281.2 M+Na] determined by ESI mass spectrometry

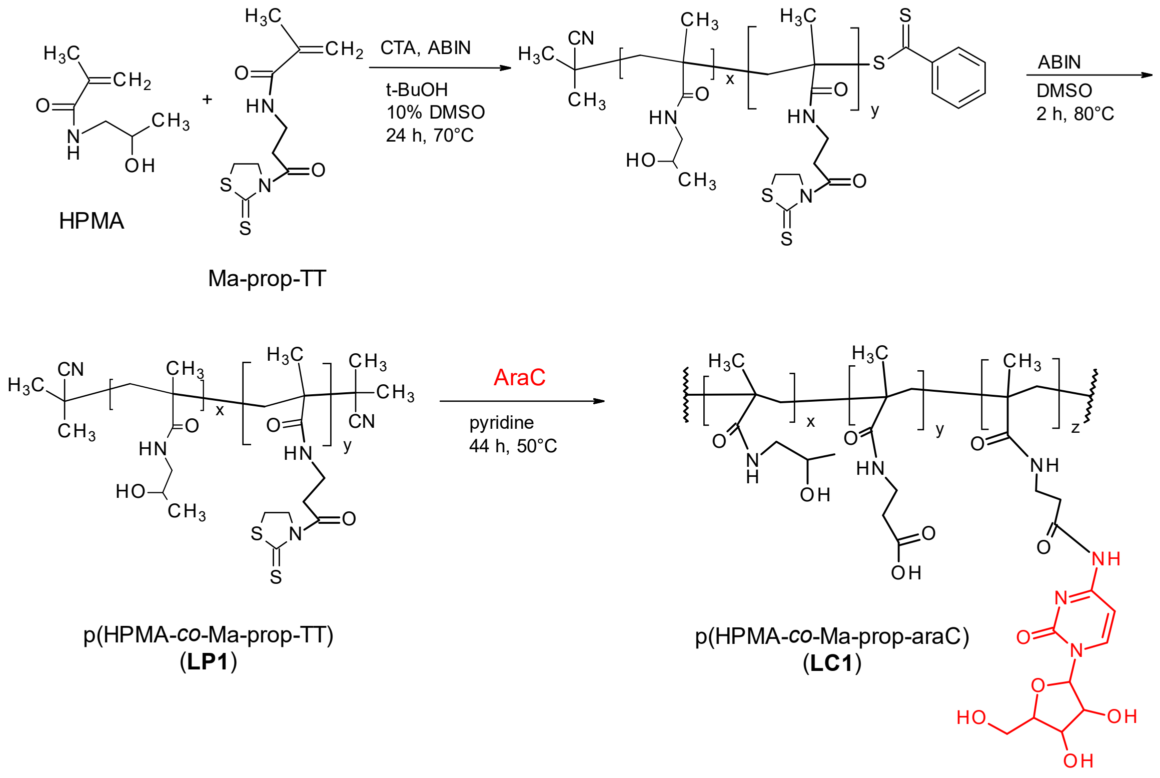

Figure S2. Scheme of polymer precursor **LP1** synthesis and formation of **LC1** conjugate through the attachment of araC

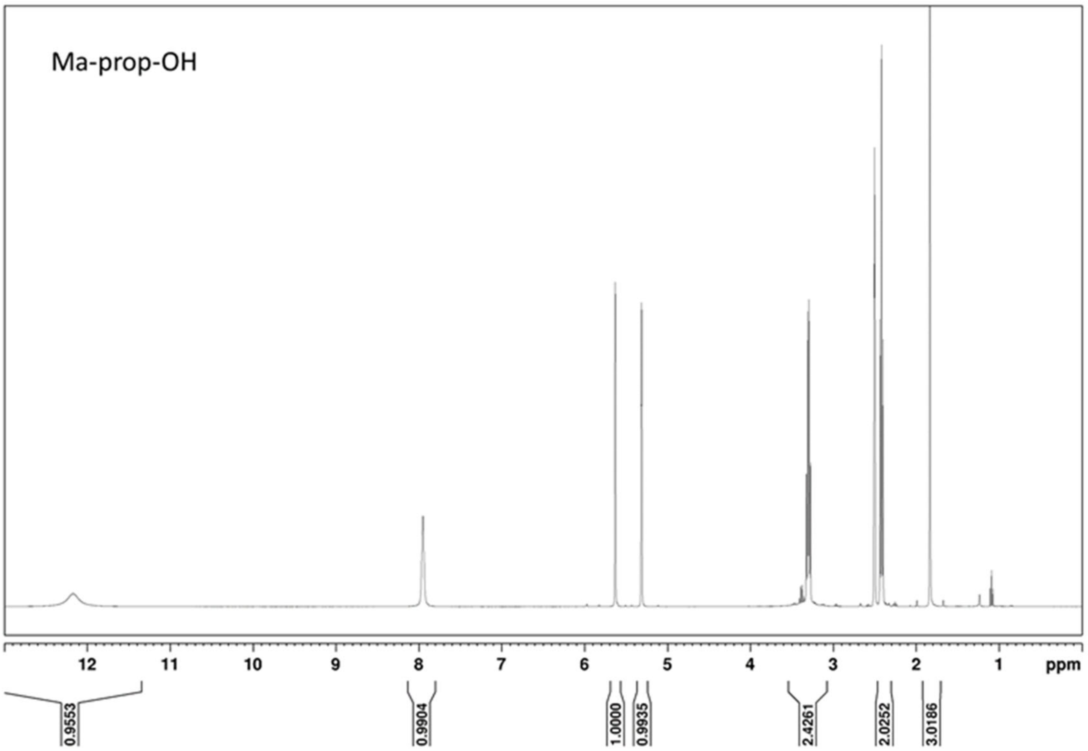
Ma-prop-OH
12 11 10 9 8 7 6 5 4 3 2 1 ppm
0.9553
0.9904
1.0000
0.9935
2.4261
2.0252
3.0186

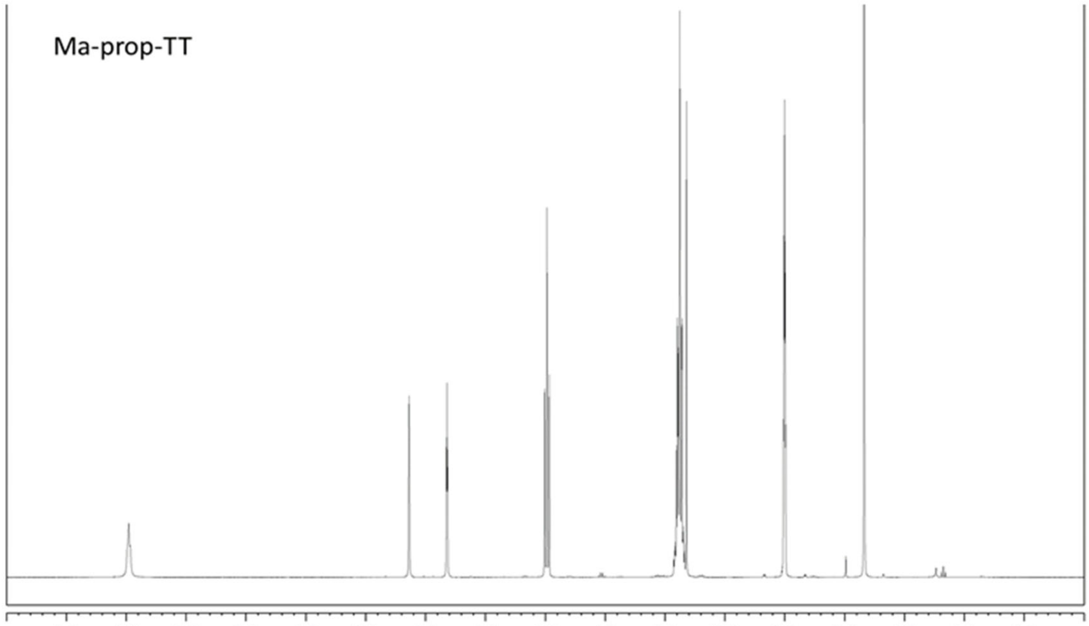
Ma-prop-TT
8.5 8.0 7.5 7.0 6.5 6.0 5.5 5.0 4.5 4.0 3.5 3.0 2.5 2.0 1.5 1.0 0.5 ppm
0.9692
1.0000
1.0018
2.0032
5.9906
3.0054

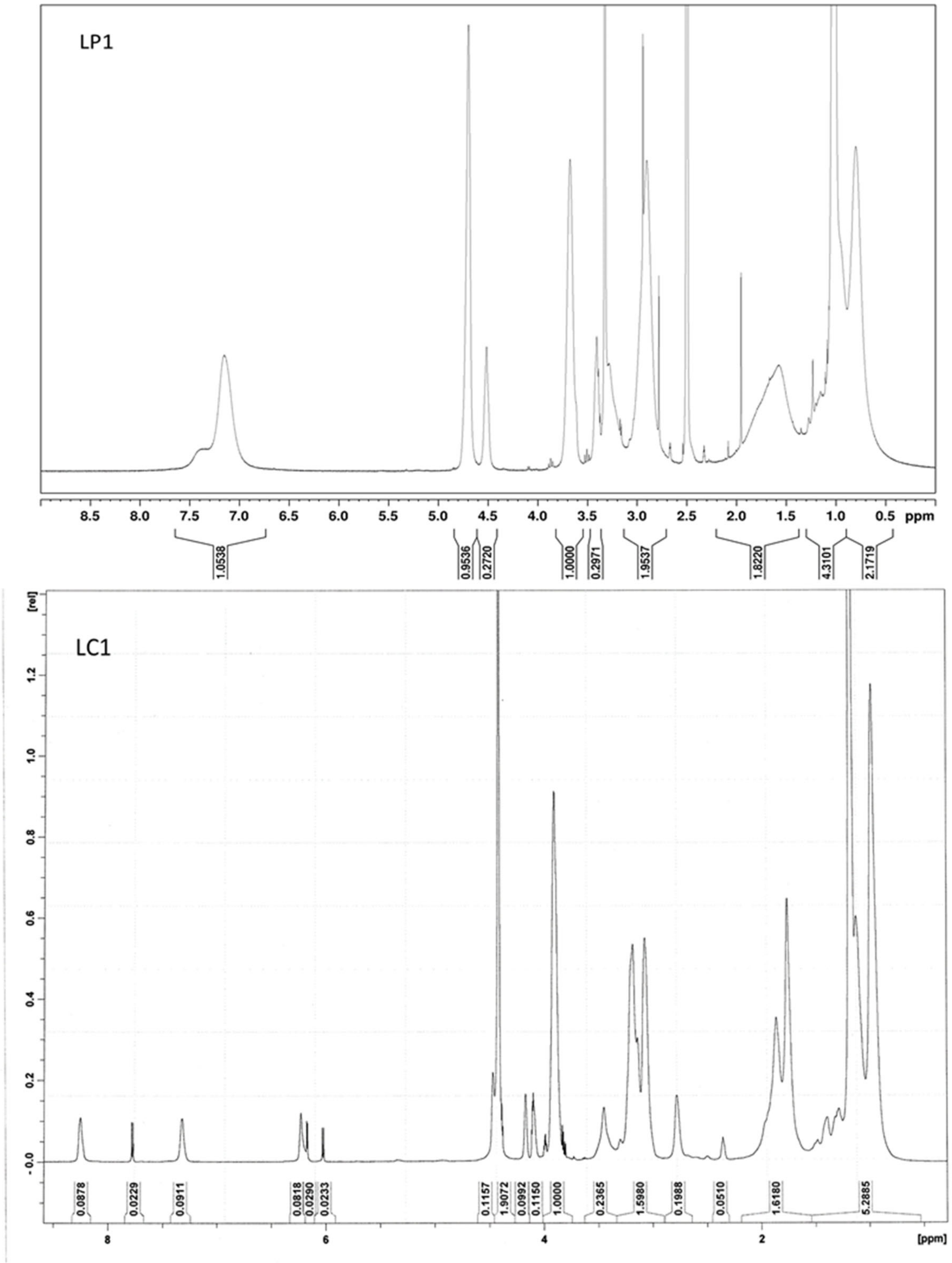

Figure S3. NMR spectra of Ma-prop-OH, Ma-prop-TT, polymer precursor **LP1** and the conjugate **LC1**.

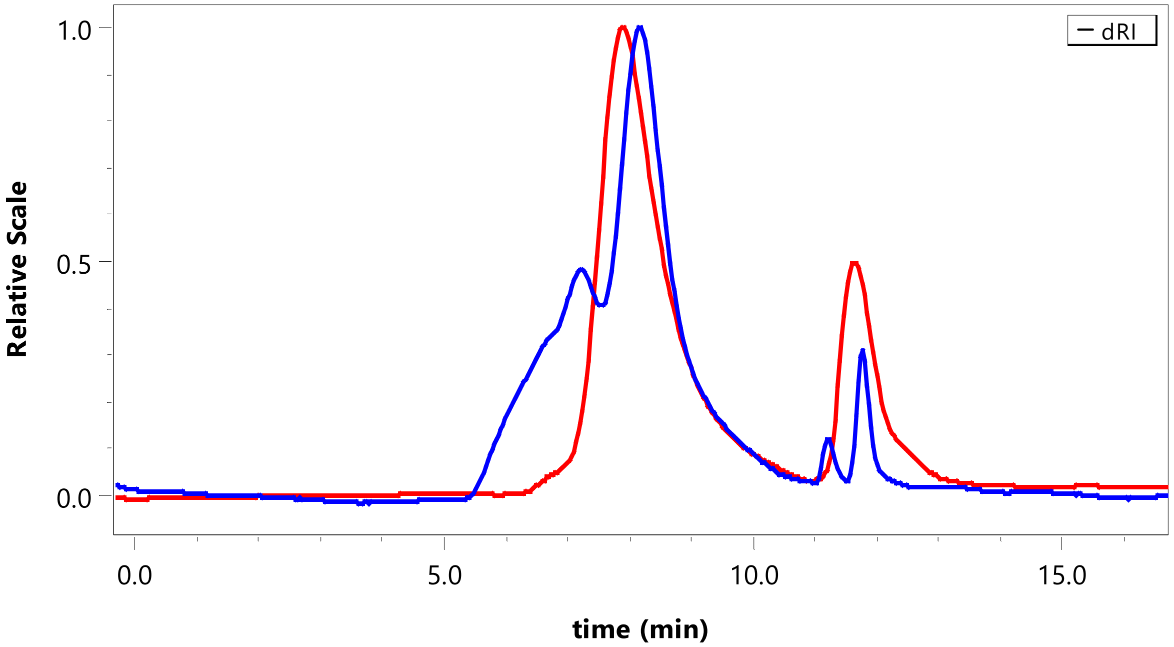

Figure S4. SEC chromatograms (differential refractive index - dRI) of **LP1** (red) and **LC1** (blue) measured on a TSK 3000 SWXL column (Tosoh Bioscience, Japan) in 80% methanol, 20% 0.3 M acetate buffer pH 6.5 at a flow rate of 0.5 ml min^{-1}.

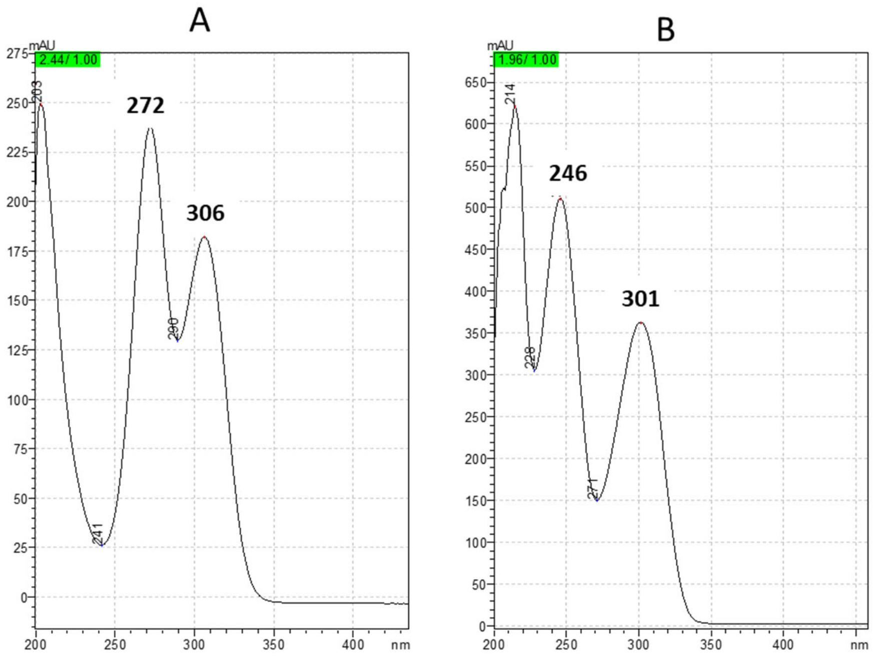

Figure S5. UV/VIS spectra of the polymer precursor **LP1** with two characteristic absorption maxima for TT at 272 and 306 nm (**A**), and conjugate **LC1** with absorption maxima at 246 and 301 nm characteristic for attached araC (**B**).

The toxicity evaluation in KTC-bearing mice

The body weight monitoring of KTC-bearing mice was performed during treatment with AraC-based linear conjugates **LC1**, **LC2**, and **LC3.** All treatment groups received the same dose (3 mg AraC equivalent/mouse), and body weights were recorded throughout the 67-day experiment, Figure S1.

The control group (black line) maintained relatively stable weight over the short period before early tumor progression occurred and mice were sacrificed due to tumor burden. Among treated groups, all the groups showed a stable body weight trend, with minor fluctuations and overall gradual gain, indicating a favorable safety profile. There was no evidence of toxicity for all of the treatment cohorts during the experiment.

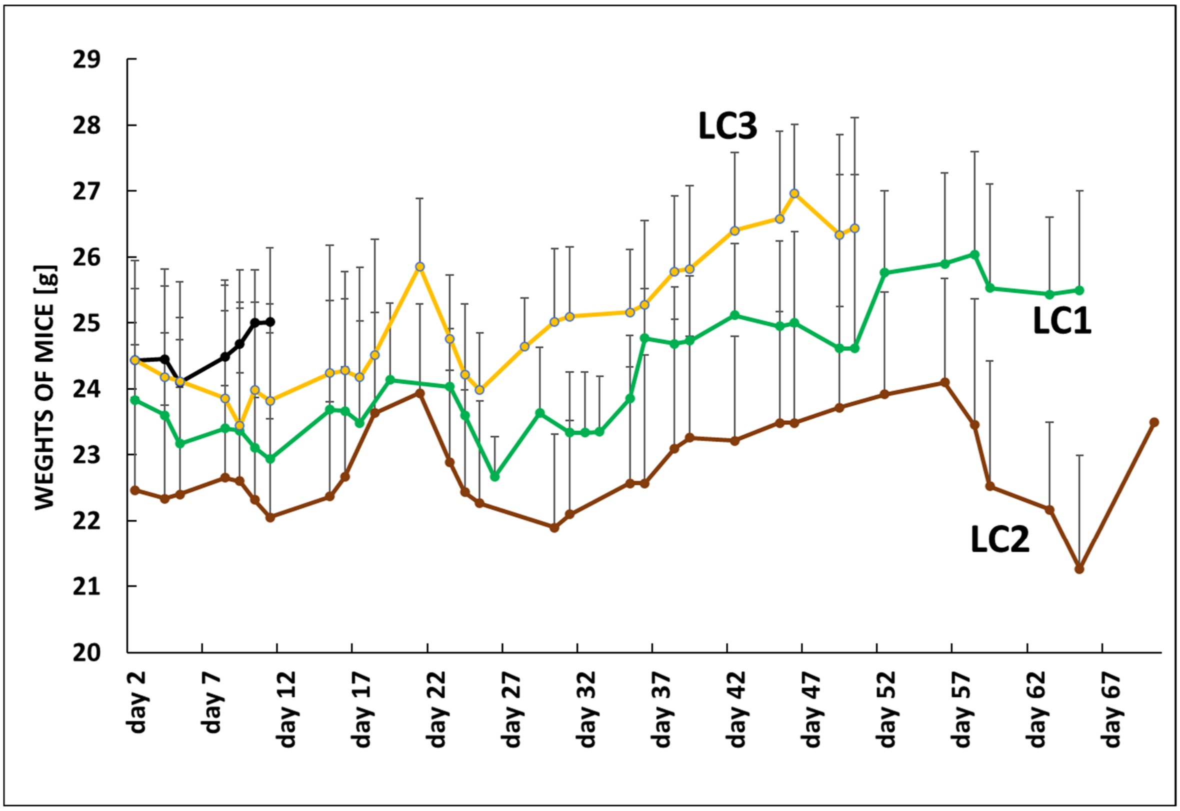

Figure S6. Weight of animals during the experimental therapy of KTC-bearing mice with linear conjugates **LC1**, **LC2** and **LC3**. Mice were xenografted with the PDX cells derived from a patient with relapsed mantle cell lymphoma (KTC). Therapy was administered IP on day 1, when all mice developed tumors with calculated volume approx. 250 mm3. Each cohort contained 5 animals. The following dosing was used: **LC1** (green line) 3 mg araC eq. /mouse, **LC2** (brown line) 3 mg araC eq. /mouse, **LC3** (orange line) 3 mg araC eq. /mouse. The results were compared to untreated animals (black line).
